# Exploring the Role of Autophagy-Related Gene 5 (*ATG5*) Yields Important Insights Into Autophagy in Autoimmune/Autoinflammatory Diseases

**DOI:** 10.3389/fimmu.2018.02334

**Published:** 2018-10-17

**Authors:** Xin Ye, Xu-Jie Zhou, Hong Zhang

**Affiliations:** Renal Division, Peking University First Hospital, Peking University Institute of Nephrology, Key Laboratory of Renal Disease, Ministry of Health of China, Key Laboratory of Chronic Kidney Disease Prevention and Treatment (Peking University), Ministry of Education, Beijing, China

**Keywords:** autophagy, ATG5, autoimmune disease, apoptosis, immunity

## Abstract

Autophagy is a highly conserved process that degrades certain intracellular contents in both physiological and pathological conditions. Autophagy-related proteins (*ATG*) are key players in this pathway, among which *ATG5* is indispensable in both canonical and non-canonical autophagy. Recent studies demonstrate that *ATG5* modulates the immune system and crosstalks with apoptosis. However, our knowledge of the pathogenesis and regulatory mechanisms of autophagy in various immune related diseases is lacking. Thus, a deeper understanding of *ATG5*'s role in the autophagy mechanism may shed light on the link between autophagy and the immune response, and lead to the development of new therapies for autoimmune diseases and autoinflammatory diseases. In this focused review, we discuss the latest insights into the role of *ATG5* in autoimmunity. Although these studies are at a relatively early stage, *ATG5* may eventually come to be regarded as a “guardian of immune integrity.” Notably, accumulating evidence indicates that other *ATG* genes may have similar functions.

## Introduction

Autophagy is a highly conserved homeostatic process from yeast to mammals. Derived from Greek “self” and “eating”, autophagy is the regulated cellular degradation of certain intracellular molecules and organelles (1). This process initiates from the engulfment of the unwanted cytoplasmic content, followed by fusion with the lysosome, and degradation. Generally, baseline (constitutive) autophagy in mammals occurs under physiological conditions, but can be increased by starvation or by various pathologies, including ischemic, toxic, immunological, and oxidative insults (2).

Autophagy is a tightly regulated process and the key players in this pathway are the AuTophaGy-related (ATG) proteins. To date, at least 41 *ATG* genes have been identified (3). The ATG core proteins are classified in five functional groups: (1) The ULK kinase (Unc-51 like autophagy activating kinase) complex (ULK1 or ULK2, ATG13, RB1CC1/FIP200, and ATG101); (2) the class III phosphatidylinositol 3-kinase (PtdIns3K) complex (BECN1/Beclin 1, ATG14, PIK3C3/VPS34, and PIK3R4/p150/VPS15); (3) the ATG12 conjugation system (ATG7, ATG10, ATG12, ATG16L1, and ATG5); (4) the microtubule-associated protein 1 light chain 3 (LC3) conjugation system (ATG4, ATG7, ATG3, WIPI2, and LC3 protein family); and (5) the ATG9 trafficking system (ATG2A and ATG2B, WIPI4, and the transmembrane protein ATG9A) (4).

Among these 41 proteins, ATG5 is indispensable for autophagic vesicle formation. Knocking down or knocking out ATG5 can result in downregulation or total inhibition of autophagy, suggesting that ATG5 plays a central role in autophagy. Thus, *ATG5* is one of the most commonly targeted genes in autophagy gene editing assays. In addition, ATG5 has other functions, including mitochondrial quality control after oxidative damage; negative regulation of the innate antiviral immune response through direct association with retinoic acid receptor responder 3 (RARRES3) and mitochondrial antiviral signaling protein (MAVS); lymphocyte development and proliferation; MHC II antigen presentation; adipocyte differentiation; and apoptosis (5).

The direct association between *ATG5* and autoimmunity was identified in hypothesis-free genome-wide association study (GWAS) data. Several GWASs for systemic lupus erythematosus (SLE) confirmed genetic associations between common variants in/near *ATG5* and SLE, in Caucasians and Asians. Similar associations were identified in other autoimmune diseases, including rheumatoid arthritis, systemic sclerosis, and multiple myeloma (6, 7). *ATG5* alleles were associated with blood pressure, insulin sensitivity, glucose homeostasis, and age-related macular degeneration using GWAS (5, 8). These data strongly supported a genetic role in the development of immune related diseases, metabolism, and cancer.

Considering *ATG5*'s intimate association with immune related diseases as introduced above, we chose ATG5 as an example to discuss the importance of autophagy in immune related diseases. We also discuss ATG5's structure, function and related phenotypes. We hypothesize that the selective restoration of ATG5 function could be used to treat systemic autoimmune diseases.

## Structure of ATG5

Formerly known as apoptosis specific protein (ASP), ATG5 was first identified in Burkitt's lymphoma apoptotic cells. *ATG5* locates to human chromosome 6q21. Several transcript variants encoding protein isoforms have been identified. It can be transcribed from an open reading frame of 828 bp in yeast cells, encoding a protein of 276 amino acids. Human ATG5 comprises 275 amino acids, with an estimated molecular weight of ~32.4 kDa. Western blotting shows a band of ~56 kDa, representing the ATG5-ATG12 complex (9).

To date, there has been no direct X-ray crystallography study of ATG5 and thus little information is available for the structure of ATG5. One major reason is that ATG5 frequently binds with other proteins to form multiprotein complex and thus the isolated form is difficult to obtain. Furthermore, ATG5 is a soluble protein, and is prone to aggregation during purification (10). However, many studies have revealed the crystal structure of complexes involving ATG5, where the crystal structure of ATG5 is also obtainable. Matsushita et al. studied the ATG5-ATG16 complex and reported that ATG5 comprises three domains, including two ubiquitin-like (Ubl) domains flanking a helix rich domain (11). The two Ubl domains (UblA and UblB) both include a five-stranded β-sheet and two α-helices, exhibiting similar structures. The helix-rich domain comprises three long and one short α-helix. These three domains fold into the unique overall architecture of ATG5, where many protein interactions take place. Understanding the structure and binding sites of ATG5 complexes is important for the further determination of its functions.

ATG5 commonly binds with ATG12, catalyzed ATG7 and ATG10 (12, 13). ATG12's C-terminal glycine residue forms a covalent conjugation with a lysine residue of ATG5, forming the ATG5-ATG12 complex (14). According to the crystallization analysis of ATG5-ATG12 in *Saccharomyces cerevisiae*, a covalent conjugation of ATG5 Lys149 and ATG12 C-terminal Gly 186 was observed (11, 14). The ATG5-ATG12 complex also has several non-covalent interactions, including hydrophilic and hydrophobic interactions (14). After ATG5 binds covalently with ATG12, the complex further interacts with ATG16 via non-covalent linkages. The ATG16 α-helix interacts with UblA, UblB, and the short helix in helix-rich domain of ATG5, and its loop interacts with ATG5 UblA exclusively (11).

Tectonic β-propeller repeat containing 1 (TECPR1) is another key component in autophagy that promotes the fusion of lysosomes and autophagosomes (15, 16). TECPR1 includes several repeating tectonin β-propeller repeats, two dysferlin domains, an internal ATG12-ATG5 interacting region (AIR) domain, and a pleckstrin homology (PH) domain. TECPR1 functions by binding to the ATG5-ATG12 complex (17). Human ATG5 interacts with TECPR1 and binds non-covalently to the two ubiquitin-fold domains and N-terminal helix α-1 (10).

As stated above, current structural analysis is restricted to ATG5 complexes, because isolated human ATG5 is difficult to obtain. In the future, a more advanced structural analysis technique, i.e., cryogenic electronic microscopy (cryoEM), might be used to analyze the detailed structure of ATG5, which will increase our understanding of the function and pathogenic mechanisms of autophagy proteins.

## Roles of ATG5 in autophagy

### Canonical autophagy

Canonical autophagy includes macroautophagy, microautophagy, and chaperone-dependent autophagy. Macroautophagy (hereafter referred to as autophagy), the classical pathway of autophagy, is initiated by the formation of an omegasome from the endoplasmic reticulum. The omegasome then forms an isolated membrane that further undergoes elongation, simultaneously engulfing intracellular components. Finally, the isolated membrane is closed as a complete autophagosome that then fuses with a lysosome, forming an autolysosome, in which the contents are degraded by lysosomal hydrolases, completing autophagy (2).

ATG5 is important in the context of autophagy (Table 1). Absence of ATG5 in mice causes neonatal lethality, possibly by disrupting autophagy and thus inhibiting the engulfment of lipid droplets (19, 44, 45). Furthermore, ATG5 can modulate autophagy. For example, under the conditions of starvation or rapamycin blockage, receptor activated C-kinase 1 (RACK1), a scaffold protein, binds with ATG5 to initiate autophagosome formation (46). The key role of RACK1-ATG5 in autophagy was further demonstrated by knockdown of RACK1, which demonstrated attenuated autophagy, and that blocking the interaction between ATG5 and RACK1 inhibits autophagy (47). Although the exact role of RACK1 is not fully understood, it is evident that RACK1 interacts with ATG5 and regulates autophagy. Another example is Calpain 1, a calcium dependent ubiquitous non-lysosomal cysteine protease that digests ATG5 (48). Low intracellular calcium levels downregulated the cleavage activity of Calpain 1, increasing the level of ATG5 and ATG12-ATG5 and ultimately upregulating autophagy (49). Thus, ATG5 levels can be regulated via Calpain 1 cleavage, which has a marked influence on autophagy. Similarly, microRNA miR181a interacts with an miRNA response element in the 3′ untranslated region (UTR) of *ATG5*, which inhibits its transcription. Overexpression of miR181a significantly attenuates *ATG5* mRNA and protein levels, resulting autophagy inhibition (50).

**Table 1:** Phenotypes of cells or organisms lacking ATG5 in different species.

**Species**	**ATG5-absent cell/organ**	**Phenotype**
L. major	Whole	Reduced flagellum, reduced virulence (18)
Mouse	Whole	Neonatal death (19, 20); more susceptible to liver fibrosis (21)
	B lymphocyte	Significant defect in B cell development at the pro- to pre-B cell transition (22); decreased antigen secretion (23); increased cell death (24)
	T lymphocyte	Increased CD8+ T cell death; decreased CD4/8+ T cell proliferation (25)
	Dendritic cell	Defect in processing and presentation of phagocytosed antigens (26)
	Macrophage	Increased plaques in artery wall (27); impaired restriction of pathogen L. Major (28)
	Embryo fibroblast	Higher level of apoptosis (29); higher migrating activity (30)
	Neuron	Progressive deficits in motor function and degeneration of some neural cells (31)
	Purkinje cell	Degenerate early and axonal swelling (32)
	Liver	Decreased survival in sepsis (33); higher hepatocyte proliferation (34)
	Lung	Alveolar epithelial cells are unable to mobilize internal glycogen stores independently of surfactant maturation (35)
	Renal proximal tubule cell	Accelerated cell death (36); G2/M arrest (37); decreased renal function (38, 39)
	Renal podocyte	Glomerular filter barrier damage, accelerated glomerulosclerosis (40)
Human	Adult-generated neuron	Reduced survival, delay in cell maturation (41)
	Cardiac myocyte	Increased ischemia/reperfusion cell injury (42)
	Fibroblast	Greater migration ability (43)

**Figure 1:**
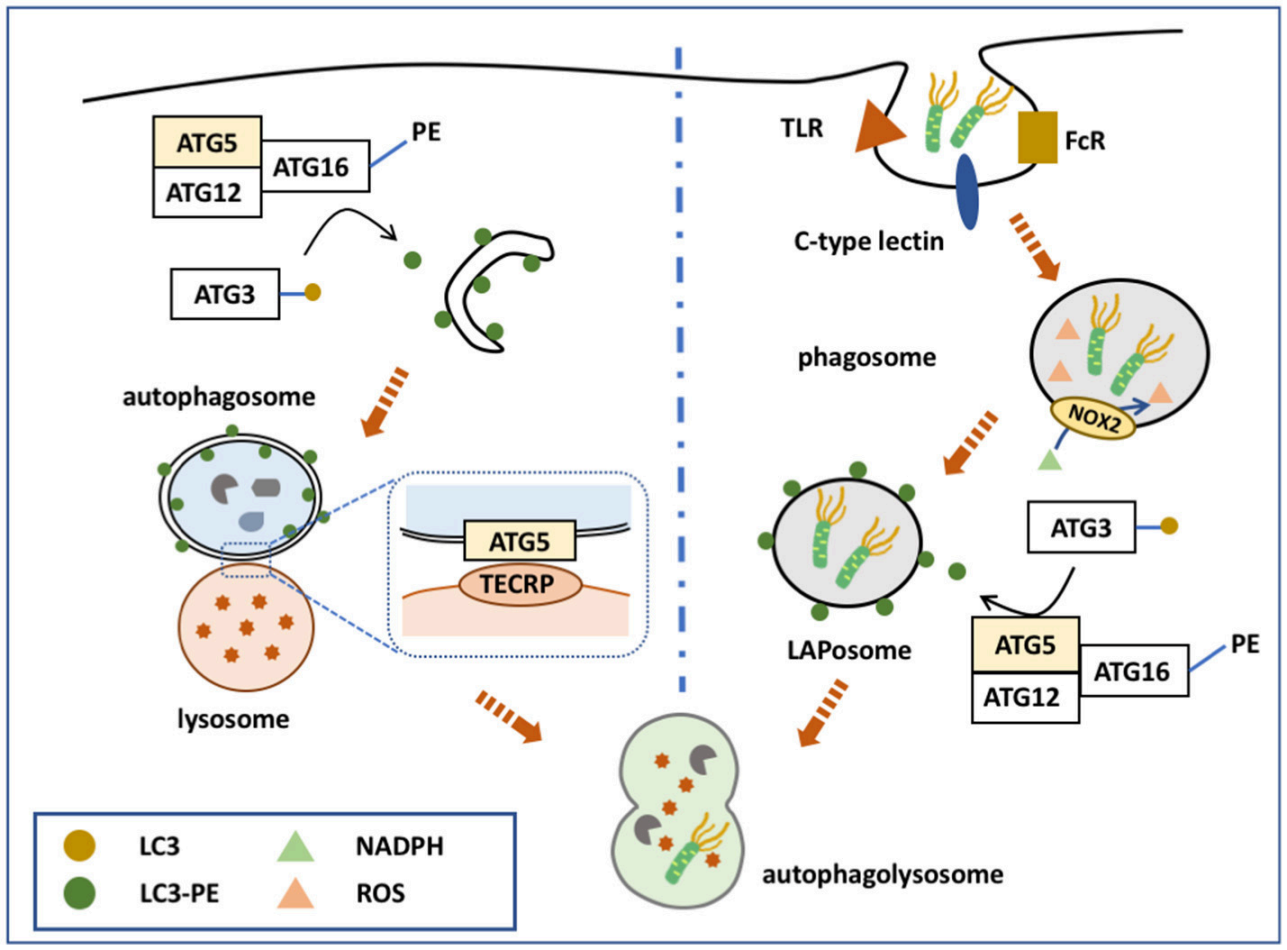
ATG5 in canonical and non-canonical autophagy. ATG5 is involved in both canonical and non-canonical autophagy processes. The left side of the figure shows how ATG5 is implicated in the process of macroautophagy. Upon binding with ATG12 and ATG16, ATG5 forms a complex to conjugate PE with LC3, which is deposited on the membrane of the autophagosome. ATG5 is also expressed on the autophagosomal membrane that binds with TECRP, and therefore promotes the fusion of autophagosomes and the lysosomes. The right side of the figure presents ATG5 involved in the process of LAP. Similarly, ATG5 helps the conjugation of LC3 and PE by forming a complex of ATG5-ATG12-ATG16, and thus accomplishes the deposition of LC3-PE on the phagosome.

The ATG5-ATG12-ATG16 complex serves as a ubiquitin-like conjugation system that contributes to the elongation of the isolated membrane and autophagosome maturation (Figure 1). The conjugation of ATG5 with ATG12 is catalyzed by ATG7 and ATG10. The complex is termed as “ubiquitin-like conjugation system” because of the similar behavior of these proteins to ubiquitin enzymes. First, ATG7 activates ATG12, which resembles an E1 ubiquitin enzyme. ATG12 is then transferred to ATG10, an E2-like enzyme, and finally conjugates to ATG5 (2). However, no E3-like enzyme for conjugating ATG12-ATG5 has been identified.

#### Membrane formation

The ATG12-ATG5/ATG16 complex helps to form the autophagosome membrane in two ways: By involving in the LC3-PE conjugation pathway, or by directly binding with the membrane. Although the LC3- phophatidylethanolamine (PE) conjugation system was discovered earlier, these two pathways have equally important roles in autophagosome membrane formation. The major function of ATG12-ATG5/ATG16 is to serve as an E3-like enzyme in another ubiquitin-like conjugation system, the LC3 system (51), which mainly links LC3 with PE, and thus is termed the lipidation conjugation system. In this system, ATG7 serves as an E1-like enzyme to activate LC3 (52). LC3 is then transferred to ATG3, another E2-like enzyme, and conjugates with PE with the help of the ATG12-ATG5/ATG16 complex (13). Although the ATG12 complex helps the conjugation of LC3-PE, each component of the complex serves different roles (53). Recent studies showed that ATG16 is essential for the efficient conjugation of LC3-PE; however, previous studies, using different methods, disagreed with this conclusion. ATG5 facilitates direct membrane binding, while ATG12 inhibits it. ATG12-ATG5/ATG16 also binds directly to the autophagosome membrane to accomplish its formation, independently of LC3. ATG5 alone is able to bind with the autophagosome membrane, while conjugation with ATG12 inhibits this binding (54). After the non-covalent linkage with ATG16, ATG5 regains its membrane binding function. ATG16 might possess a coiled-coil domain that functions to dimerize ATG5-localized membrane binding sites (55). This hypothesis is supported by the observation that ATG5 alone cannot induce clustering of the membrane, while the ATG5/ATG16 complex can form giant unilamellar vesicles (54). Furthermore, inactivation of the ATG5-ATG12/ATG16 complex inhibits autophagosome formation to a greater extent than inhibiting LC3-PE conjugation (56–59).

#### Fusion

ATG5-ATG12 is also involved in autophagosome-lysosome fusion. TECPR1, localized on the lysosomal membrane, binds with the ATG5-ATG12 complex to regulate autophagosome-lysosome fusion (16). When not involved in autophagy, the TECPR1 AIR domain occupies the PH domain to interrupt its binding with the autophagosome membrane (16). However, during autophagy, ATG5-ATG12 is generated to bind with the TECPR1 AIR domain, making the PH domain available to bind with phosphatidylinositol 3-phosphate of the autophagosomal membrane. TECPR1 is located on the lysosome membrane and ATG5 is localized on the autophagysomal membrane; therefore, TECPR1 binding with ATG5-ATG12 induces autophagosome and lysosome fusion (15, 60).

### Non-canonical autophagy

Non-canonical autophagy is characterized by either generating autophagosomes without the macroautophagy pathways, or involves canonical autophagy pathways without autophagosome formation. Currently, several pathways are identified in non-canonical autophagy, including LC3-associated phagocytosis (LAP); Beclin-1 independent autophagy; autophagosome formation from multiple phagophores and pathogen-specific autophagy modification; autophagy-associated unconventional protein secretion; and defective ribosomal products-containing autophagosome-rich blebs (61).

During pathogen-associated molecular pattern (PAMP) receptor activation [e.g., Toll-like receptor (TLR), Fc receptor, and C-type lectin], a PI3PK complex is recruited to the phagosomal membrane (62–64). Unlike in macroautophagy, this complex lacks ATG14, but consists of Rubicon and UVRAG (65). Subsequently, NADPH oxidase 2 (NOX2) is recruited to the phagosome with the help of Rubicon. PI3PK initiates lipidation of the phagosomal membrane, while NOX2 is responsible for the production of reactive oxygen species (ROS) (66). Together, these two events activate the two conjugation system to deposit LC3-PE on the outer membrane of the phagosome, forming a LAPosome. Thereafter, the LAPosome fuses with the lysosome, and LAPosome constituents are degraded. Complete LAPosomes may also fuse with endosomal vesicles, including major histocompatibility complex (MHC) class II-containing compartments, to present peptides to T helper cells (67).

ATG5, via its lipidation of LC3, plays a key role in the two conjugation systems of the LAP pathway (Figure 1). It is speculated that LC3 lipidation might alter the activity of lysosome fusion. Indeed, several studies demonstrated that ATG5 could regulate the fusion of LAPosomes and lysosomes by initiating LC3 lipidation. During TLR activation, phagosomes ligated with LC3-PE exhibited more rapid fusion with lysosomes compared with LC3-free phagosomes (64). Moreover, knockdown of ATG5 inhibited this promoting effect, suggesting that ATG5-dependent LAP promotes the fusion process (64). Consistently, blocking LAP by knocking down ATG7 attenuated the fusion between LAPosomes and lysosomes, while LC3 recruitment to the phagosome is suggested to accelerate its maturation and fusion (68, 69).

However, other studies suggested that the deposition of LC3-PE was a delaying factor during fusion. LC3-PE-positive phagosomes are longer lived and mature later. Delayed phagosome-lysosome fusion also leads to prolonged MHC II antigen presentation. Other studies focused on the relationship between antigen presentation and ATG5-dependent LC3-PE deposition on phagosomes, which confirmed the delaying effect of LC3-PE on fusion. Lysosomal protease activity was related to the internalized antigen degradation speed (70). Macrophages with higher levels of lysosome protease maintained intracellular antigens for a shorter time. This suggested that delaying phagosome and lysosome fusion could delay the degradation of the antigens inside the phagosome. This finding was confirmed by another study demonstrating that ATG8/LC3-positive phagosomes could prolong antigen presentation, while Atg5-deficient macrophages failed to present extracellular antigens onto MHC class II molecules (63). Prolonged antigen presentation resulted in longer or continuous CD4+ T cell activation, and a more permanent humoral immune response.

## ATG5 in immunity regulation

Studies suggest that the major roles of autophagy in the immune system include elimination of microbes, control of inflammation, lymphocyte homeostasis, and the secretion of immune mediators. Thus, it is reasonable to infer that ATG5 could regulate certain aspects of the immune system, which has been confirmed by extensive research. In this section, we simply summarize the roles of ATG5 in innate and adaptive immunity, including regulating immune cell activation, cytokine secretion, and pathogen secretion.

### Innate immunity

In response to different stimuli, macrophages can be polarized into proinflammatory M1 or anti-inflammatory M2 (71). ATG5 regulates autophagic activity to alter the polarization of macrophages, subsequently modifying the extent of inflammation. ATG5 knockout hepatic macrophages hyperpolarized to the M1 phenotype, and therefore secreted more cytokines [interleukin (IL)-6 and tumor necrosis factor (TNF)] to increase the inflammatory response (72). Thus, ATG5-dependent autophagy is responsible for regulating macrophage polarization.

ATG5 activates neutrophils indirectly. In the presence of ATG5, lipopolysaccharide stimulates the secretion of mitochondrial proteins and autophagosomal luminal proteins, further activating polymorphonuclear leukocytes (73). Thus, lipopolysaccharide (LPS)-stimulated extrusion of mitochondrial contents provokes an inflammatory response of immune cells in an ATG5-dependent manner.

**Figure 2:**
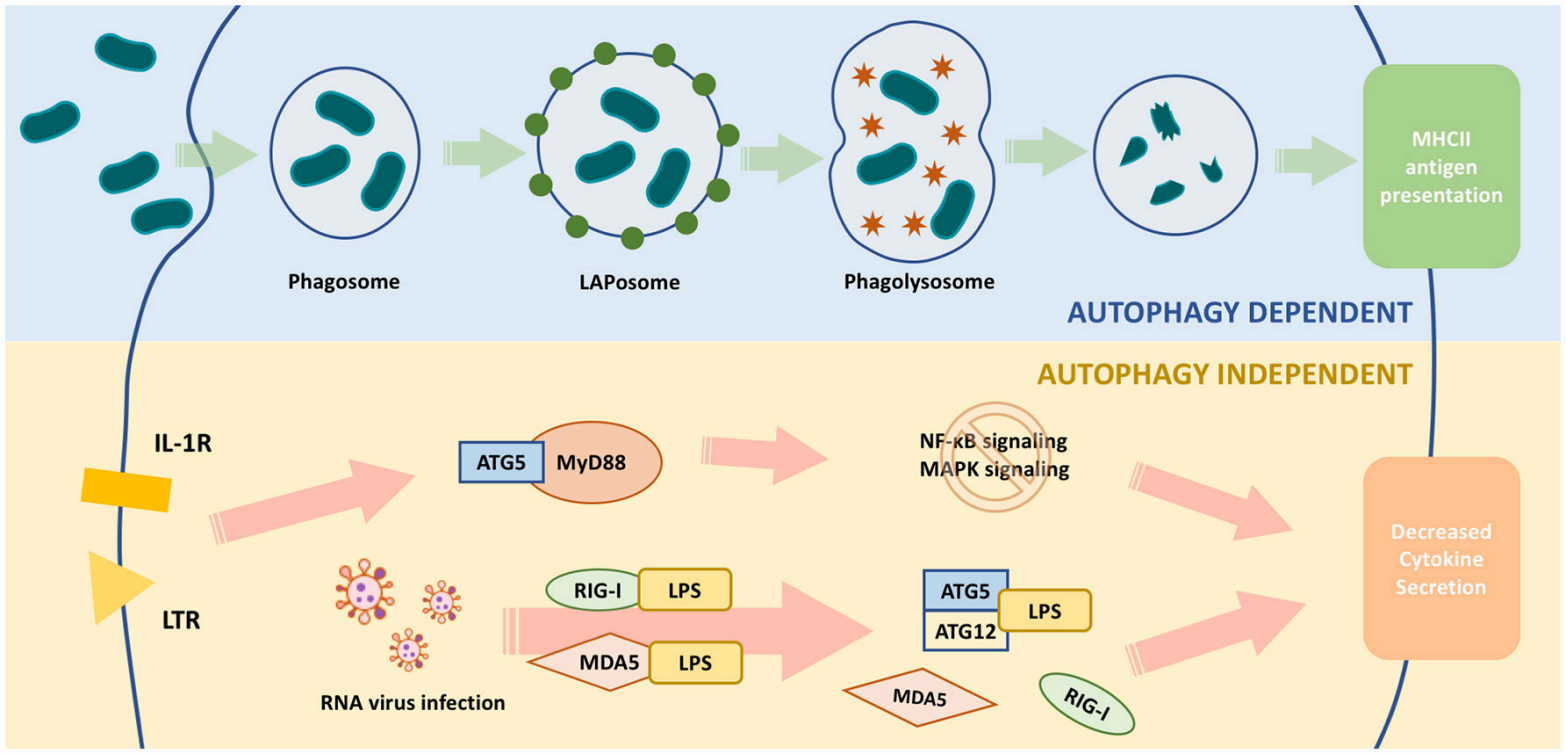
Functions of ATG5 in macrophage. The upper part of the figure illustrates ATG5, as an important part of autophagy, is involved in the process of pathogen clearance and antigen presentation. The lower part illustrates ATG5 regulates macrophage cytokine secretion in an autophagy-independent manner. During RNA virus infection, ATG5-ATG12 binds with IPS to block the conjugation of IPS with RIG-1 or MDAS, and finally inhibits the expression and secretion of IFN. When TLR or IL-1R is activated, the downstream pathways, such as NF-κB signaling and MAPK signaling, are activated through MyD88. ATG5 is able to bind with MyD88 to block these pathways and eventually attenuates the production of certain cytokines.

ATG5 regulates MyD88-dependent signaling to regulate innate immune responses (Figure 2). MyD88 is an important signaling adaptor molecule for TLRs and the IL-1 receptor, ultimately activating nuclear factor (NF)-κB signaling and mitogen-activated protein kinase (MAPK) signaling cascades, which leads to the transcription of many genes involved in innate and adaptive immunity (74, 75). ATG5 interacts with MyD88 and interrupts the downstream pathways, thereby suppressing NF-κB signaling (76). Thus, ATG5-mediated NF-κB signaling suppression might be involved in immune regulation.

The growth of intracellular bacteria can be restricted by autophagy, either canonical or non-canonical, in which ATG5 is essential. *Mycobacterium tuberculosis* is one of the pathogens in vacuoles that is eliminated by autophagy (77) and a double membrane structure was observed in tuberculosis infected type II alveolar epithelial cells (78). Atg5 knockout mice presented with a heavier *M. tuberculosis* burden, more severe inflammation, and higher levels of IL-1 (79). Autophagy also targets cytosolic bacteria, such as Group A Streptococcus (GAS). Mouse embryonic fibroblasts infected with GAS presented GAS-containing autophagosome-like vacuoles, while ATG5-deleted cells failed to produce such structures (80).

Recently, ATG5-mediated restriction of microbial infection via LAP was confirmed, and silencing or inactivation of ATG5 inhibited LAP activity and increased the survival of pathogens, including adherent and invasive *Escherichia coli, Shigella flexneri, M. tuberculosis, Aspergillus fumigatus*, and HIV (81–83). In particular, MORN2 recruits LC3 in macrophages to eliminate *M. tuberculosis* infection, and ATG5 is implicated in the process (84). Moreover, certain pathogens, such as *S. flexneri*, could interact with ATG5 to interrupt LAP and evade elimination. By binding with IcsB, an *S. flexneri* effector, ATG5 failed to bind with IcsA, another effector, thereby halting the LAP process and the elimination of the pathogen (85). However, recruiting ATG5 to promote LAP does not always help pathogen clearance. After HIV-1 infection, phagocytosis of, Vpu recruits ATG5 and LC3 to promote fusion with lysosomes to accelerate the degradation of the HIV-1 capsid protein, and thus favoring the dissemination of HIV into the cell (86).

Intriguingly, ATG5 also eliminates pathogens in an autophagy-independent manner. ATG5 regulates cytokine secretion through crosstalk with various pathways, and ATG5-mediated cytokine secretion achieved elimination of the pathogens. In addition, ATG5 recruited IFN-γ-inducible p47 GTPase IIGP1 (Irga6), which triggered IFN-γ-mediated clearance of *Toxoplasma gondii* (87). However, classical characteristics of autophagy, such as autophagosomes enveloping *T. gondii*, were not detected, further proving the autophagy-independent nature of ATG5-mediated *T. gondii* clearance. Similarly, *Atg5*-deleted mice vaginal cells expressed lower levels of cytokines involved in the anti-*Candida albicans* response, resulting in a lower *Candida* clearance rate (88).

### Adaptive immunity

**Figure 3:**
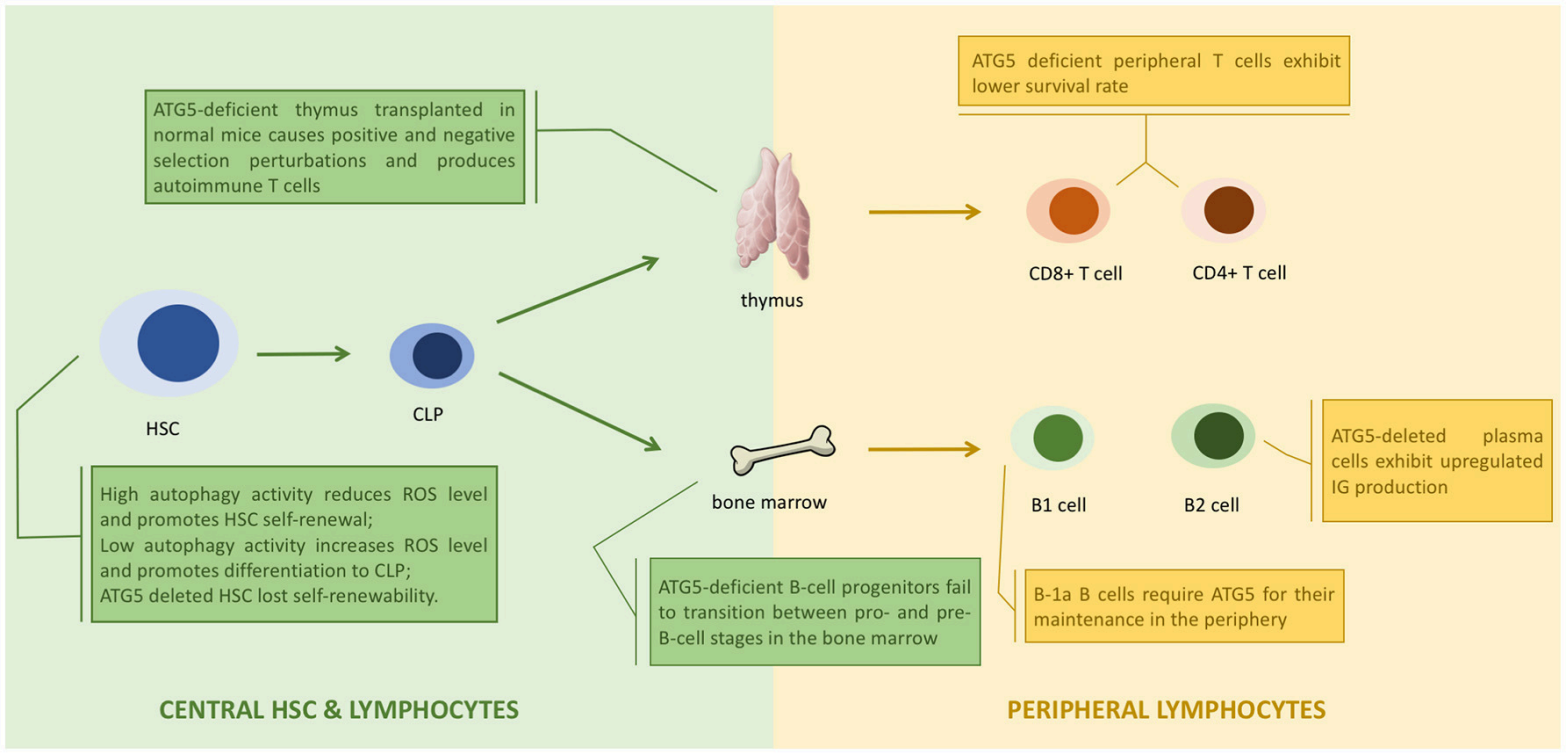
ATG5 in the development of lymphocytes. ATG5 regulates the proliferation and differentiation of lymphocytes in each stage (1). Autophagy regulates the status of hematopoietic stem cell (HSC) through modulating the level of reactive oxygen species (ROS). Deletion of ATG5 in HSC inhibits autophagy and results in loss of self-renewability (2). ATG5 regulates lymphocyte maturation via autophagy in both bone marrow and thymus. Loss of ATG5 in this stage results in maturation failure and autoimmunity (3). ATG5 is responsible for the homeostasis of peripheral lymphocytes. Deletion of ATG5 in peripheral lymphocytes exhibit cell potency like autoimmunity.

ATG5 assists antigen presentation through autophagy, and thus is responsible for indirect lymphocyte activation by promoting the interaction between T or B cells and antigen presenting cells (APCs) (89). ATG5 is also directly responsible for regulating lymphocytes. ATG5-deleted CD8+ T lymphocytes were prone to cell death, and ATG5-deleted CD4+ and CD8+ T cells failed to undergo efficient proliferation after T-cell receptor (TCR) stimulation (90). The decreased survival of ATG5-deleted T cells was caused by the accumulation of abnormal autophagic structures and dysregulation of mitochondrial and ER homeostasis (25). ATG5-deleted progenitors failed to successfully transit from pro- to pre-B-cells. Knocking out Beclin-1 in B cells also resulted in differentiation failure (22). Thus, ATG5 might regulate lymphocyte development in an autophagy-dependent manner (Figure 3).

During RNA virus infection, retinoid acid-inducible gene I (RIG-I) or melanoma differentiation associated gene 5 (MDA5) are activated to bind with interferon-β promoter stimulator 1 (IPS-1) through their caspase recruitment domains (CARDs), eventually upregulating the production of type I interferon (91, 92). Type I interferon is an important cytokine responsible for enhancing antigen presentation and activating certain subtypes of immune cells, such as natural killer cells, cytotoxic T cells, B cells, and memory T cells (93). The ATG5-ATG12 complex bound with the CARD of RIG-I or MDA5 to inhibit the production and the secretion of interferon (94). Thus, ATG5 could regulate the production of type I interferon and the elimination of RNA viruses by influencing adaptive immunity activity.

In summary, ATG5 is responsible for the activation and the differentiation of various immune cells in innate and adaptive immunity. Evidence suggests that ATG5 regulates these immune cells via autophagy.

## ATG5 in cell death

**Figure 4:**
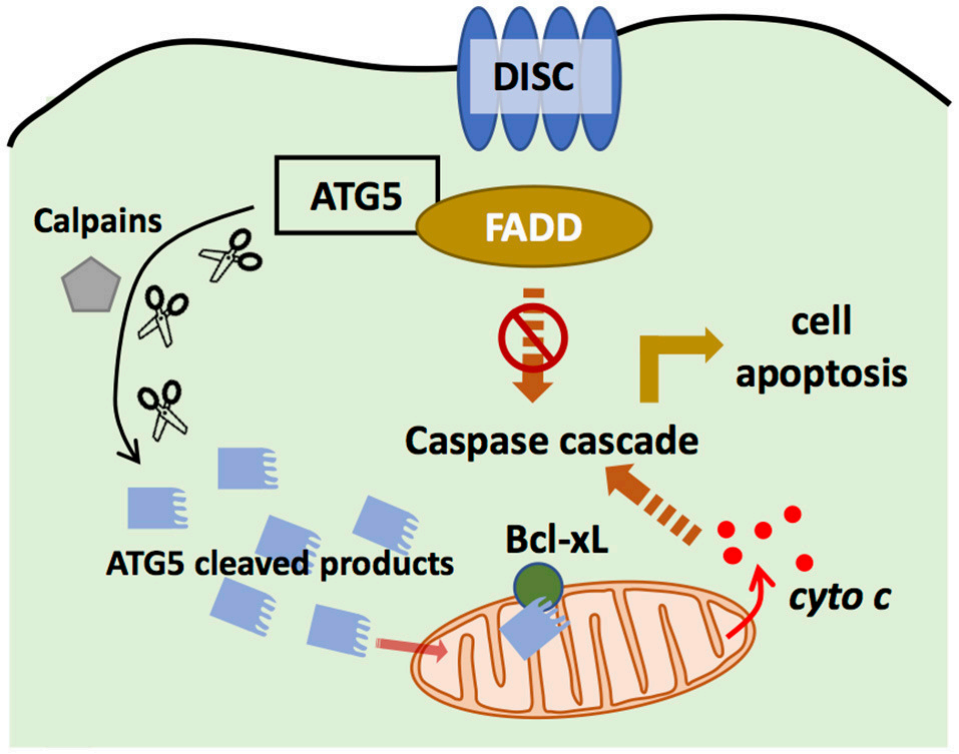
ATG5 in apoptosis. ATG5 is able to bind with Fas associated protein with death domain (FADD) to block the binding of death-induced signaling complex (DISC) and FADD, and thus inhibits extrinsic apoptosis. ATG5 can also be cleaved by Calpains. The cleaved products then are translocated to the mitochondria to bind with Bcl-xL, and promote the secretion of cytochrome c, which activates intrinsic apoptosis.

Apoptosis is a programmed cell death process (95), whose pathways are distinct according to different stimulations (96). DNA damage generally triggers the intrinsic apoptosis pathway, where Bax and Bak induce the secretion of cytochrome c, leading to apoptosis (97, 98). Death receptor activation triggers the extrinsic apoptosis pathway, where a death-induced signaling complex (DISC) is formed to bind with Fas associated protein with death domain (FADD), leading to apoptosis (99, 100). By contrast, autophagy is regarded as a cytoprotective process in cell survival. Interestingly, evidence demonstrates crosstalk between autophagy and apoptosis (Figure 4).

ATG5 can regulate the extrinsic apoptosis pathway. ATG5 could bind with FADD to interrupt the interaction between FADD and DISC, halting the extrinsic apoptosis pathway (101). However, downregulation of ATG5 did not influence FADD-dependent cell death, and inhibiting caspase, the key component in apoptosis, did not block autophagosome formation (102), suggesting that apoptosis and autophagy are distinct processes. However, recent studies have challenged this conclusion. Yousefi et al. provided direct evidence that an apoptosis-related protease cleaves ATG5 to regulate the apoptosis and autophagy balance. Calpain mediated N-terminal cleavage product of ATG5 makes several different cell types more responsive to apoptotic stimuli. Apoptosis is associated with the translocation of this ATG5 fragment from the cytosol to mitochondria, in which it associates with the anti-apoptotic molecule Bcl-xL and triggers cytochrome c release and caspase activation, without activating autophagy (48). Similarly, the administration of Trichokonin VI (TK VI), an antimicrobial peptide, triggered the influx of extracellular calcium, which induced calpain-mediated ATG5 cleavage (103). TK VI administration also generated ROS, whose accumulation damages mitochondria, leading to ATG5-dependent autophagy (103). Thus, ATG5 has an important role in the crosstalk between autophagy and apoptosis.

ATG5 is considered to exert its function as a key component in autophagy, which takes place in the cytoplasm, where ATG5 is commonly localized. However, ATG5 is also localized and functions in the nucleus. *ATG5* expression is upregulated after DNA damage, and ATG5 is recruited to the nucleus through a leucine-rich nuclear export signal (NES) (104). Inside the nucleus, ATG5 competes with aurora kinase B (AURKB) to bind with survivin, which inhibits the formation of the chromosome passenger complex responsible for chromosome segregation (105, 106). Failure to generate the chromosome passenger complex causes mitotic catastrophe, an oncosuppressive phenomenon occurring during or after defective mitosis, leading to death or senescence, and eventually resulting in G2/M arrest and the cessation of mitosis (107). This nuclear role of ATG5 in mitotic catastrophe shed it to be another spotlight in a separate form of cell death.

In summary, ATG5 is responsible for crosstalk among different forms of cell death. ATG5 also interrupts mitosis and promotes cell death triggered by DNA damage. Apoptosis is implicated in the pathogenesis of several autoimmune diseases, and thus we cannot rule out that the association of ATG5 with certain diseases is solely linked with autophagy or apoptosis (108). Further research is needed to determine whether apoptosis plays a role in ATG5-induced autoimmune diseases.

## ATG5 and autoinflammatory disease

The term “autoinflammatory” was coined in 1999 to define a newly discovered family of recurrent fever syndromes (109). Compared with autoimmune diseases, autoinflammatory diseases are characterized by a lack of provocation for inflammation and the absence of high-titer autoantibodies or antigen-specific T lymphocytes (110). In the pathogenesis of the traditionally defined autoimmune diseases, such as SLE, antigen receptor rearrangement and mutation play significant roles (111). In contrast, autoinflammatory diseases mainly involve aberrant innate immunity instead of adaptive immunity. During the last decade, genetic studies have identified a large number of gene mutation loci associated with abnormalities of innate immunity in several autoinflammatory diseases. Among them, several genes related to autophagy affect innate immunity associated with the development of autoinflammatory diseases. The following section introduces two autoinflammatory diseases, Crohn's disease and type 2 diabetes mellitus, and their association with autophagy.

### Crohn's disease (CD)

Crohn's disease is a non-specific chronic inflammatory disorder of the gastrointestinal tract. Its pathogenesis has several mechanisms, although the exact process remains unclear. CD is not traditionally defined as an autoimmune disease; however, its development involves the immune system attacking certain organs of the body. Therefore, CD is reasonably regarded as an “autoinflammatory” disease (112). Similar to SLE, the development of CD involves the interaction of genetic predisposing factors and environmental stimulation. The basis of CD pathophysiology is a classical Th1 cell reaction, in which TNF-α has a central role (113).

GWAS studies revealed that *ATG16L1* and immunity-related GTPase family M (*IRGM*) polymorphisms increase CD susceptibility (114–118), leading to extensive exploration of the association between autophagy and CD pathogenesis. IGRM initiates autophagy to eliminate invasive pathogens (119). Deletion of IRGM in human intestinal epithelial cells and macrophages caused defective autophagy (120, 121). Moreover, a CD-related *IRGM* single nucleotide polymorphism (SNP) (rs10065172, c.313C>T) produces an *IRGM* variant that fails to bind with miR-196, resulting in defective autophagy and attenuated pathogen clearance (122). In addition, miR-196 is overexpressed in the inflammatory intestinal epithelia of patients with CD, confirming that IRGM might play a role CD pathogenesis of CD by interfering with autophagy (123). It is observed that exposure to microbial products or bacterial invasion increases IRGM expression. And IRGM physically interacts with 2 other CD risk factors, ATG16L1 and NOD2, and additional pattern recognition receptors such as NOD1, RIG-I, and select TLRs. This explains how polymorphisms altering expression or function of autophagy in pathogenesis of infection and CD.

The *ATG16L1* risk allele causes defective lysozyme secretion of intestinal Paneth cells and autophagy dysfunction in intestinal macrophages, resulting in pathogen elimination failure (124). Previous studies showed that Paneth cells from mice carrying *Atg16l1*^T300A^ cannot secrete lysozyme through secretory autophagy when infected with *S. typhimurium*. Mice with selective ablation of autophagy in intestinal epithelial cells (IECs) (*Atg16l1*^VC^ mice) exhibit severely exacerbated intestinal pathology, characterized by increased accumulation of CD4^+^ T cells in the lamina propria and elevated levels of pro-inflammatory cytokines. *Atg16l1*-deficient IECs show increased induction of apoptosis following exposure to pro-inflammatory cytokines (TNF + IFNG/IFNγ) compared to wild-type IECs. These findings confirm that the exacerbated pathology in *Atg16l1*^VC^ mice is largely driven by TNF-induced IEC apoptosis (125, 126). More recent data also suggested an interaction between smoking and *ATG16L1*^T300A^ triggers Paneth cell defects in Crohn's disease (127).

ATG16L1 contributes to autophagy by forming the ATG5-ATG12-ATG16 complex; therefore, it unsurprising that similar morphological abnormalities are observed in ATG5 deleted Paneth cells, which were indistinguishable from ATG16L1 deleted cells (128). GWAS did not identify any CD pathogenesis-related *ATG5* SNPs; however, a prospective pharmacogenomic study of patients with CD treated with anti-TNF-α drugs reported several significant SNPs in *ATG5* and *ATG12* as associated with positive response to therapy (129). In addition, ileal biopsy samples of patients with CD revealed an inverse correlation between levels of microRNAs miR30C and miR130A and those of ATG5 and ATG16L1 (121). The possible mechanism was revealed by *in vitro* experiments, showing that inhibiting the two microRNAs in cultured mice intestinal epithelial cells upregulated the expression of *Atg5* and therefore restored autophagy function (128). Downregulation of ATG5 by miRNAs, leading to defective autophagy and inflammation, might be involved in the pathogenesis of CD.

ATG5 is associated with maintaining the regular functioning of Paneth cells and intestinal macrophages. Despite GWAS showing that *ATG5* is not directly related, *ATG5* is associated with CD patients' response to therapy, suggesting that it might serve as a downstream player in CD pathogenesis or some certain phenotypes. However, further studies are needed to determine the detailed mechanism of ATG5 in the development of CD.

### Type 2 diabetes mellitus

Diabetes is an extensively investigated disease, characterized by elevated serum glucose, which might result in internal organ damage when not carefully controlled, such as diabetic nephropathy and diabetic retinopathy. Type 1 diabetes (T1DM) is an autoimmune disease, whose pathogenesis involves the production of pancreatic β cell antibodies, while type 2 diabetes (T2DM) centers around the state of insulin resistance (IR). However, inflammation and abnormal cytokines secretion from fat tissues are believed to play a major role in the development of IR, placing it in the spectrum of “autoinflammatory” diseases. Recent studies explored the relationship between ATG5 and IR, implying a possible role of ATG5 in T2DM development.

Significant evidences links obesity and T2DM with autophagy, where ATG5 plays a certain role disease development. Ultrastructural analysis of adipose tissue (AT) adipose tissue in obese and T2DM patients revealed increased numbers of autophagosomes and increased immunofluorescence signal of marker LC3 (130–132). Furthermore, several autophagy markers, including ATG5, are increased in visceral AT as well as subcutaneous AT of obese and T2DM patients, suggesting ATG5-dependent autophagy might be involved in the development of obesity-induced T2DM (130). Moreover, increased activity of ATG5-dependent autophagy is also linked with higher TNF-α and IL-6 expression, suggesting that autophagy serves as a pro-inflammatory factor to enhance AT inflammation (130). In addition, levels of autophagy marker genes were increased in insulin resistant compared with insulin sensitive patients (132). ATG5 also regulates pancreatic β cell homeostasis by regulating autophagy (133). A substantial amount of proinsulin is rapidly delivered to autophagosomes and directed to lysosomal degradation, and deletion of ATG5 results in increased proinsulin, suggesting that ATG5-dependent autophagy might play a critical role in the production and secretion of insulin (133). Thus, ATG5 is responsible for regulating insulin production homeostasis in pancreatic β cells and for enhanced inflammation and IR in AT, revealing the possibility that ATG5 is closely related to the pathogenesis in T2DM.

However, ATG5 does not always appear to exacerbate the development of T2DM. The administration of dihydromyricetin (DHM), a natural flavonoid that exerts various bioactivities, including anti-oxidative effects, attenuated IR severity by promoting AMPK-induced autophagy, which also upregulated ATG5 (134). Similarly, AMPK signaling was also activated in amepelopsin-treated endothelial cells, which triggered ATG5-dependent autophagy (135).

Although there is some evidence demonstrating the involvement of ATG5 in T2DM, its exact role in the development of T2DM remains unclear. Whether the increase in ATG5 in certain tissues has as a protective role from inflammation and IR requires further research.

## ATG5 and autoimmune diseases

Given the multi-faced function of ATG5, it is reasonable to speculate that it might be involved with other diseases whose pathogenesis interferes with autophagy or apoptosis; for example, the large spectrum of autoimmune diseases. Genetic predisposition and environmental stimulation both contribute to disease development. Generally, autoimmune diseases are characterized by immune cells or molecules attacking tissues or organs of the human body, resulting from false activation of immune cells by “self-derived” components. Therefore, aberrant autophagy or apoptosis might expose intracellular contents to the matrix, which could activate immune cells to trigger an autoimmune response.

### Systemic lupus erythematosus

Systemic lupus erythematosus (SLE) is a systemic autoimmune disease that affects multiple organs, including the skin, muscles, joints, kidney, and heart (136). The etiology of SLE is complex and not fully understood. Infection, UV exposure, certain drugs, and imbalanced hormone levels are risk factors for SLE that would undermine the immune system and provoke autoimmunity (136). Aberrant autoimmunity in SLE includes defects in clearing apoptotic cells, and abnormal antigen presentation and autoantibody production (137). These autoantibodies can directly target organs or form immune complexes to further damage tissues (137).

Studies have shown that mammalian target of rapamycin complex 1 (mTORC1) inhibition increases autophagy, whereas stimulation of mTORC1 reduces this process. And it have revealed that mTORC1 represses autophagy by phosphorylating and repressing ULK1 and ATG13. Activation of the mTOR pathway might induce abnormalities in lymphocytes of patients with SLE (138, 139). Depletion of glutathione, and increased nitric oxide and mitochondria in T cells are observed in patients with SLE (140–142). Consistently, low glutathione and high nitric oxide trigger mTOR signaling, which subsequently induced persistent mitochondrial hyperpolarization (MHP), presenting as a mitochondrial mass in T cells (140–142). Moreover, mTOR activation upregulates the expression of small GTPases to promote the recycling of TCR-associated signaling proteins (143). In patients with SLE, small GTPase-dependent lysosome degradation of CD3, a TCR-associated signaling protein, is observed, suggesting activation of the mTOR pathway (144). Therefore, autophagy might play an important role in SLE pathogenesis via mTOR signaling.

Moreover, ATG5 is implicated in SLE through LAP. Mice with *Atg5*-deleted myeloid cells exhibited LAP deficiency. Repeated injection of dying cells into these LAP-deficient mice induced the development of an SLE-like disease, including increased serum levels of autoantibodies and creatinine. Intriguingly, knocking out other autophagy genes, *Atg14* and *Fip200*, undermined canonical autophagy but not LAP, and did not induce SLE-like disease, which suggested that ATG5-dependent LAP plays a definite role in the pathogenesis of SLE.

The genetic association between *ATG5* alleles and SLE provides strong evidence of the role of autophagy in SLE. Several GWASs have identified SNPs in *ATG5* that are genetic predisposing factors for SLE. The first GWAS study (2008) reported the association of *ATG5* with SLE in females with European ancestry. Further evidence showed that *ATG5* SNPs are related to SLE in Caucasian and Chinese populations (145–148). However, other studies identified important SNPs in the intergenic region of *PRDM1*-*ATG5*, such as rs548234 and rs6568431. Moreover, GWAS from a Chinese population reported the association between SLE and *PRDM1*-*ATG5* instead of *ATG5*, which further obscured the role of ATG5 in SLE (147). Thirty-one genes involved in NF-κB signaling, IFN and IL-12 production, and apoptosis pathways are regulated by ATG5 genotypes (148). In addition, several SNPs, including rs548234, rs693612, rs9480642, rs6937876, rs548234, and rs6937876, exhibiting significant correlations with ATG5 expression [*cis* and *trans*- expression quantitative trait loci (eQTLs)] were also associated with SLE susceptibility. In addition, further follow-up study also suggested that rare variants (mutations) apart from SNPs were also associated with SLE (149). And SNPs that affect ATG5 expression (ATG5-trans eSNPs) also showed genetic associations with SLE (150). By *in-silico* analysis, all these associated SNPs were regulatory SNPs for ATG5 expression, suggesting a significant role of deregulated ATG5 expression in mediating SLE. And it was indeed observed ATG5 was increased in patients with SLE. A pilot study also observed significant gene-gene interactions between *ATG5, ATG7*, and *IRGM* (148). And a genetic pathway based study not only confirmed these associations, but also identified novel associations with LC3 (148). Of note, most genetic studies focusing on autophagy were mainly conducted in Chinese populations, thus more wide-spread replications are still warranted.

ATG5 acts on the immune system to accelerate the inflammatory response, including NF-κB and interleukin production, and functions in antigen presentation. SLE-related *ATG5* SNPs influence these key pathways; therefore, it is reasonable to speculate that ATG5 initiates the development of SLE by disrupting antigen presentation or causing a cytokine imbalance. Tested genotypes of *ATG5* also showed changes in apoptosis-related protein expression, which also revealed ATG5's role in apoptosis in the pathogenesis of SLE.

A more recent study observed that the administration of shATG5-lentivirus ameliorated proteinuria and decreased the level of serum anti-dsDNA antibody in lupus-prone mice, suggesting promising therapeutic innovations targeting ATG5; however, more investigation is needed to evaluate its side effects (151).

### Central nervous system (CNS) autoimmunity

Central nervous system autoimmune diseases comprise a large spectrum of diseases, each of which requires extensive research and investigation. Recent studies have revealed the role of autophagy and CNS autoimmunity, in which ATG5 might play an important role. ATG5 in dendritic cells is regarded as a possible autoimmune response driver, according to a study in which *Atg5*-deleted mice exhibited lower degree of demyelination in the CNS (152). In the absence of ATG5, CD4+ T cell presentation of endogenous myelin peptides was inhibited, which restricted the downstream autoimmune response (152, 153).

Multiple sclerosis (MS) is an autoimmune disease characterized by spatial and temporal dissemination (154). The pathophysiology of MS lies in the demyelination of the white matter of the CNS. Patients with MS present with intermittent episodes of neurological dysfunction (155). The exact mechanism of MS is not completely understood; however, an autoimmune reaction is regarded as the central mechanism. CD4+ Th1 cell dependent cell-mediated immunity is suggested as the dominant autoimmune reaction damaging the white matter. Activated T cells, along with certain B cells, cross the blood brain barrier and provoke inflammation that induces demyelination. Meanwhile, macrophage, IFN γ, and TNF-α are also involved in the process of demyelination (156).

Experimental autoimmune encephalomyelitis (EAE) is a widely used animal model of MS, in which elevated ATG5 mRNA levels are detected in blood and brain tissue (157). The ATG5 level also correlates positively with EAE clinical severity, suggesting a possible role of ATG5 in inflammatory demyelination. The ATG5 mRNA level is elevated in patients with active relapsing-remitting MS (RRMS) compared with those in quiescent RRMS (158). Strong ATG5 immunoreactivity is also observed in postmortem brain tissue of patients with secondary progressive MS (158). However, the involvement of ATG5 in MS is obscured by a gene analysis showing that MS is not associated with *ATG5* variants (159). Therefore, to further investigate ATG5 function in MS demyelination, the post-translational state of ATG5 was analyzed. The level of the ATG5-ATG12 complex increased significantly in EAE mice, while the level of cleaved ATG5 was lower than that in control mice, which possibly represents a pro-survival role of ATG5 in T cells by enhancing autophagy and blocking apoptosis (158). The role of ATG5 in autophagy in MS was supported by the altered expression of BECN1 and LC3, two autophagy pathway components, in blood from patients with MS (160).

Neuromyelitis optica (NMO) is a CNS autoimmune disease that is associated with ATG5. Similar to MS, the major characteristic pathology of NMO is demyelination; therefore, ATG5 might exert the same function in the pathogenesis of NMO. In a Chinese Han population, *ATG5* variants were found to be associated with NMO, among which SNP rs548234 increased susceptibility, while rs548234 and rs6937876 have protective roles in NMO (159).

Evidence supporting ATG5's association with MS or NMO is limited. Elevated levels of Atg5 in MS animal models and postmortem brain tissue might not suffice to conclude that ATG5 is responsible for the pathogenesis of MS. Likewise, further investigation is needed to provide direct evidence of the underlying mechanism of how ATG5 variants result in abnormal demyelination and the development of MS and NMO.

## Conclusion and perspectives

ATG5 is an extensively investigated protein, most characteristics of which, including its gene, structure and functions, are gradually unveiling its mysterious mask. ATG5 initiates the formation of the autophagosome membrane and the fusion of autophagosomes and lysosomes, functioning in both canonical and non-canonical autophagy. ATG5 also functions in the immune system, regulating innate and adaptive immune responses, including macrophage polarization, cytokine secretion, antigen presentation, and the activation of certain immune-related cells. ATG5 is also involved in both intrinsic and extrinsic apoptosis. Lastly, ATG5 can also translocate into the nucleus and induce mitotic catastrophe. Based on its multi-faceted function, ATG5 could not only relate to MS, NMO, and SLE, as traditionally defined autoimmune diseases, but also shows association with CD and T2DM, which were considered as diseases related to autoinflammation recently.

However, our concept of the functions of ATG5 might be incomplete, and many details are lacking. In terms of the known and speculated the functions, some conclusions were simply drawn based on observing the phenotypes of ATG5 deletion or overexpression. Similarly, several diseases, such as SLE and CD, are associated with ATG5 according to GWAS, yet there have been few studies examining the exact functional role of ATG5 in these diseases. While the importance of ATG5 has only emerged, it is possible ATG5 might eventually be regarded as a “guardian of immune integrity”. An improved mechanistic understanding of the autophagy machinery could lead to treatments for human diseases. However, it is essential to further investigate the molecular mechanism of ATG5 in disease development and in executing certain functions, allowing the development of potential therapeutic innovations targeting ATG5. Notably, accumulating evidence also indicates that other ATG genes (i.e., ATG16L1, ATG7, and IRGM) may have similar functions. Further investigations are required to facilitate mechanism, biomarker and novel therapeutic intervention findings.

## Author contributions

XY collected data and conceived and wrote the manuscript. X-JZ conceived and revised the manuscript critically for important intellectual content, supervised the research group, and has given the final approval of the version to be published. HZ revised the manuscript critically, supervised the research group, and has given the final approval of the version to be published.

### Conflict of interest statement

The authors declare that the research was conducted in the absence of any commercial or financial relationships that could be construed as a potential conflict of interest.
